# Case Report: A novel deep intronic *MFSD8* splice-altering variant causes CLN7 disease through pseudoexon inclusion

**DOI:** 10.3389/fgene.2026.1980180

**Published:** 2026-10-20

**Authors:** Daria Akimova, Anait Voskanyan, Anna Orlova, Mikhail Skoblov

**Affiliations:** Department of Functional Genomics, Research Centre for Medical Genetics, Moscow, Russia

**Keywords:** CLN7 disease, deep intronic variant, MFSD8, pseudoexon inclusion, RNA analysis, splicing, whole-genome sequencing

## Abstract

Pathogenic deep intronic variants are an underrecognized cause of inherited disorders because they are typically missed by exon-focused sequencing and often require functional validation. We describe a patient with classic CLN7 disease caused by a previously unreported deep intronic variant in *MFSD8*. The patient presented with developmental regression, progressive epilepsy, cerebellar ataxia, and cerebral and cerebellar atrophy. Trio whole-genome sequencing identified a homozygous *MFSD8* variant, NM_001371596.2:c.999-556C>G, absent from population databases and previous reports. SpliceAI predicted activation of cryptic splice sites leading to inclusion of a 98-nt pseudoexon. RNA analysis of patient-derived fibroblasts confirmed aberrant pseudoexon inclusion, resulting in a frameshift and premature termination codon, with the aberrant transcript accounting for 84% of total *MFSD8* transcripts. A minigene assay independently reproduced the splicing defect, confirming that the variant disrupts normal *MFSD8* splicing. This study expands the mutational spectrum of *MFSD8* and provides functional evidence supporting the pathogenicity of a novel deep intronic splice-altering variant. Our findings further demonstrate the value of combining whole-genome sequencing with transcript analysis for the molecular diagnosis of unsolved rare neurogenetic disorders.

## Introduction

The neuronal ceroid lipofuscinoses (NCLs), collectively known as Batten disease (Bose and Tripathy, 2024), comprise a clinically and genetically heterogeneous group of neurodegenerative lysosomal storage disorders characterized by the accumulation of autofluorescent storage material within lysosomes (Mole, 2024). Disease onset typically occurs in infancy or childhood, with progressive vision impairment, motor and cognitive dysfunction, epilepsy, and premature death (Zhang et al., 2025; Ziółkowska et al., 2025). Estimated incidence ranges from 1 to 4 per 1,000,000 live births, varying by genetic subtype and population (Williams, 2011).

Historically, the NCLs were classified according to age at onset and the ultrastructural morphology of the storage material, for example, infantile (INCL), late infantile (LINCL), juvenile (JNCL), and adult (ANCL) forms. However, with the advent of gene-focused therapeutic strategies, a classification based on the underlying gene defect, augmented by age of presentation, has become more clinically relevant (Kohan et al., 2011; Williams and Mole, 2012). Among these, CLN7 disease (OMIM #610951), also known as variant late-infantile NCL (vLINCL), is caused by biallelic defects in the *MFSD8* gene. This gene encodes the major facilitator superfamily domain-containing protein 8 (MFSD8, also referred to as CLN7 protein) - a 518-amino acid lysosomal transmembrane protein whose precise function remains incompletely understood but is essential for normal neuronal homeostasis. Current evidence suggests that MFSD8 may function as a chloride channel involved in lysosomal enzyme trafficking (Zhang et al., 2025). Classic CLN7 disease typically presents between ages 2 and 7 years with seizures, motor impairment, progressive mental regression, myoclonus, speech impairment, vision loss, and personality disorders (Topçu et al., 2004).

The mutational spectrum of *MFSD8* includes missense, nonsense, frameshift, splice-site, and copy-number variants; however, pathogenic deep intronic variants remain exceptionally rare and are likely underrecognized because they are not detected by routine exon-focused sequencing approaches. To date, the deep intronic variant c.998 + 1669A>G in a compound heterozygous state with the missense variant c.929G>A (p.Gly310Asp) has been reported only in a patient with inherited retinal degeneration (Poncet et al., 2022), whereas no deep intronic variant at this locus has been associated with CLN7 disease. Because the functional consequences of noncoding variants cannot usually be inferred from DNA sequencing alone, transcript-level studies are often required to establish pathogenicity (Dirix et al., 2023; Kremer et al., 2017). The clinical relevance of such variants has been underscored by the development of milasen, a patient-specific antisense oligonucleotide targeting aberrant pseudoexon (PE) inclusion in *MFSD8*, which demonstrated the therapeutic potential of splice-correcting strategies in CLN7 disease (Kim et al., 2019).

Here, we report a patient with a novel deep intronic *MFSD8* variant causing PE inclusion, and we provide comprehensive clinical, molecular, and functional characterization of this variant, thereby expanding the mutational spectrum of CLN7 disease and highlighting the diagnostic utility of whole-genome sequencing combined with RNA analysis.

## Materials and methods

Detailed descriptions of all materials and methods used in this study are provided in the Supplementary Material section.

## Results

### Clinical findings

The proband is the first and only child of healthy, non-consanguineous Russian parents. She was born at 38 weeks of gestation after an uncomplicated pregnancy except for first-trimester threatened miscarriage. Birth weight was 3,170 g, length 52 cm, and Apgar scores were 8 and 8. Early motor and speech development were initially within normal limits, with independent walking at 13 months and phrase speech by 2 years 4 months. At approximately 4 years of age, subtle cognitive and behavioral changes, including reduced attention and memory, became apparent. At 4 years 10 months, she experienced her first atonic seizure during a febrile illness, followed by recurrent afebrile atonic seizures and later myoclonic seizures beginning at 5 years 2 months. Progressive intention tremor, gait ataxia, dysarthria, and motor incoordination subsequently developed. From the age of 6 years, rapid neurological deterioration was observed, including loss of expressive speech, regression of fine and gross motor skills, loss of bladder and bowel control, inability to walk independently by 6.5 years, and later inability to sit and feed herself independently.

Neurological examination at 6 years 8 months revealed severe developmental regression, absent speech, generalized hypotonia with brisk deep tendon reflexes and ankle clonus, marked intention tremor involving the upper limbs, head, and trunk, truncal ataxia, impaired fixation, and complete loss of independent ambulation. She was underweight (21 kg, BMI −2.9 SD) and had acquired microcephaly (head circumference 50 cm, −1.1 SD), despite tall stature for her age (height 132 cm, +2.64 SD). Brain MRI demonstrated diffuse cerebral and cerebellar atrophy (Figure 1A). Video-EEG monitoring demonstrated diffuse background slowing with generalized spike/polyspike-and-slow-wave discharges and a posterior photoparoxysmal response during intermittent photic stimulation (Figure 1B). Ophthalmological examination revealed only subtle optic disc pallor without clinically significant retinal degeneration at the time of evaluation.

**FIGURE 1:**
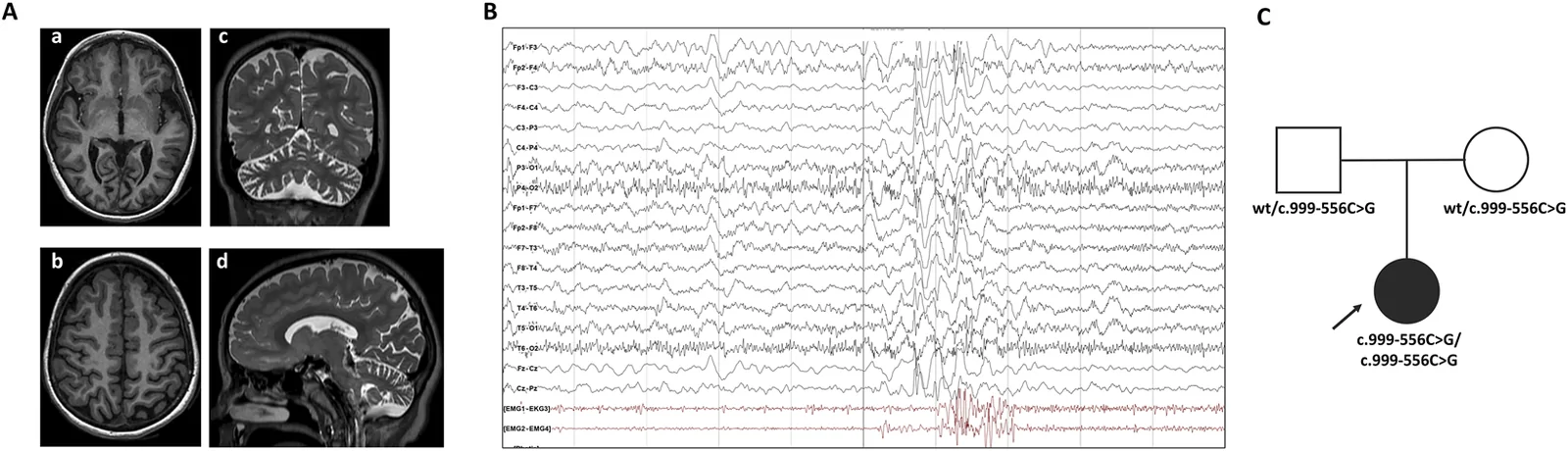
Neuroimaging and electroencephalographic findings in the proband. **(A)** Brain MRI demonstrating diffuse cerebral and cerebellar atrophy. (a, b) Axial T1-weighted images show generalized cerebral atrophy with widening of the cortical sulci. (c) Coronal T2-weighted image demonstrates cerebellar atrophy. (d) Sagittal T2-weighted image confirms diffuse cerebral and cerebellar volume loss. **(B)** Representative EEG recording showing a generalized epileptiform discharge associated with a myoclonic event. **(C)** Pedigree demonstrating the segregation of the c.999-556C>G variant within the proband’s family.

By 8 years 2 months of age, the disease had progressed further. The patient became bedridden, lost the ability to swallow independently, required gastrostomy feeding, and developed severe sleep disturbances requiring pharmacological treatment. Epileptic seizures became more frequent and could only be terminated with injectable midazolam.

Routine metabolic investigations, lysosomal enzyme assays, echocardiography, nerve conduction studies, serum creatine kinase analysis, and whole-exome sequencing were unremarkable.

### Functional characterization of the *MFSD8* variant

Trio genome sequencing subsequently identified a previously undescribed homozygous deep intronic *MFSD8* variant, NM_001371596.2:c.999-556C>G, inherited from heterozygous parents (Figure 1C). The variant is absent from gnomAD v4.1.1 and has not been reported in ClinVar or the literature. Given the proband’s clinical phenotype consistent with neuronal ceroid lipofuscinosis and the location of the variant in the *MFSD8* gene associated with CLN7 disease, functional analysis was required to confirm its pathogenicity.

SpliceAI predicted that the *MFSD8* NM_001371596.2:c.999-556C>G variant creates cryptic acceptor (delta score 0.73) and donor (delta score 0.67) splice sites, resulting in inclusion of a 98-nucleotide PE. To validate this prediction, RNA was isolated from cultured skin fibroblasts of the proband and her heterozygous father. The region spanning exons 6–12 was amplified by RT-PCR and analyzed by targeted next-generation sequencing (NGS). RNA from a healthy donor fibroblast culture served as a control.

Targeted NGS confirmed inclusion of the predicted 98-nucleotide PE (chr4:127,922,520–127,922,617) (Figure 2A). Two transcript isoforms were identified in both the proband and her father: the canonical transcript and an aberrant transcript containing the PE. Inclusion of the PE resulted in a frameshift and generation of a premature termination codon, p.(Ile334Valfs*113). The PE-containing transcript represented 84% of the MFSD8 transcript molecules detected in the targeted amplicon from the homozygous proband, compared with 12% in the heterozygous father. These values describe the relative proportions of the detected transcript isoforms and are compatible with reduced representation of the mutant transcript in the heterozygous state, potentially due to nonsense-mediated decay.

**FIGURE 2:**
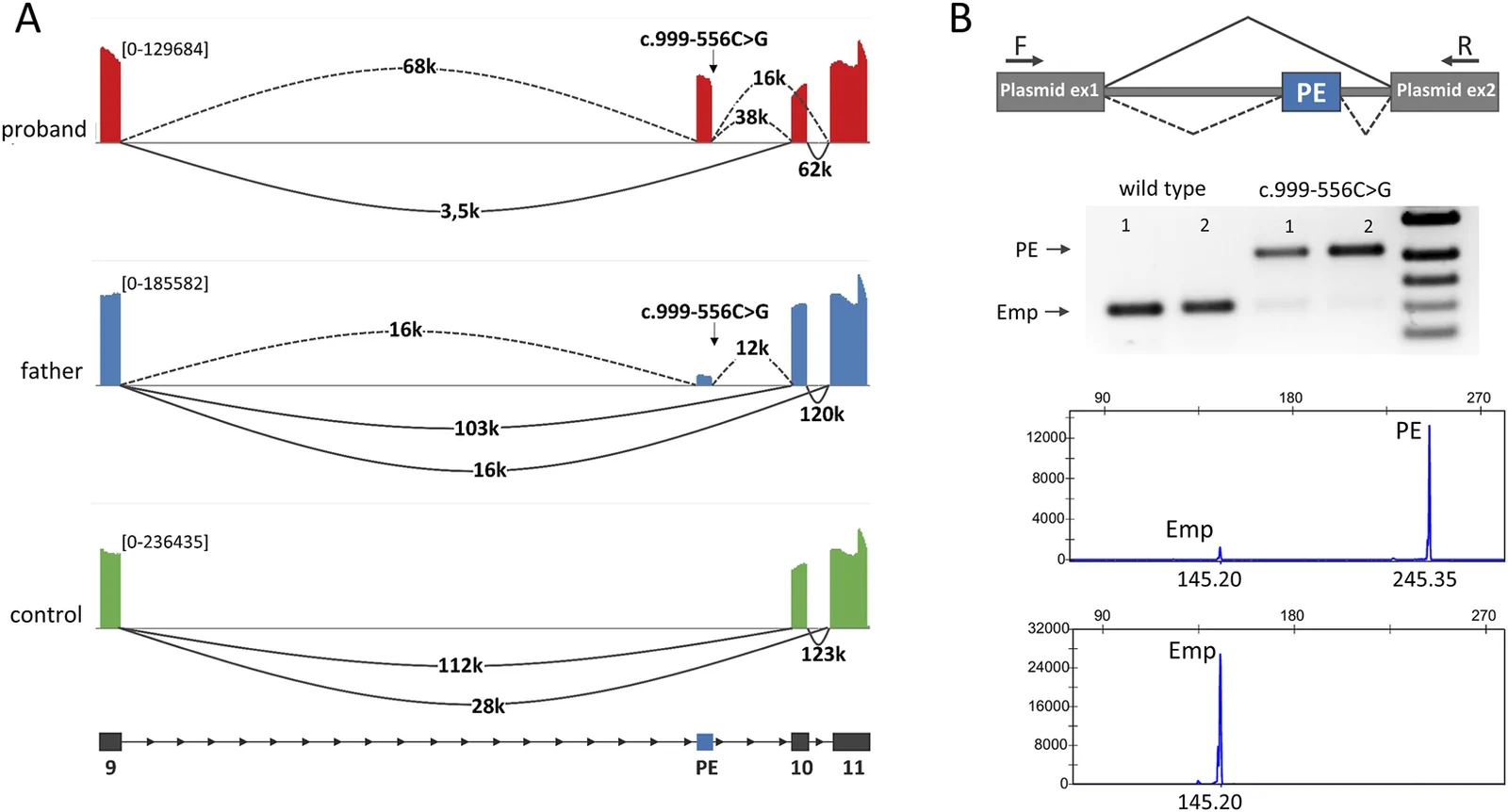
Functional validation of the deep intronic *MFSD8* c.999-556C>G variant. **(A)** Targeted RNA sequencing demonstrating inclusion of a 98-nt PE in transcripts from the homozygous proband and, at lower levels, the heterozygous father, while only the canonical transcript was detected in the healthy control. The position of the c.999-556C>G variant and the PE are indicated. **(B)** Minigene assay confirming PE inclusion induced by the c.999-556C>G variant. Top: schematic representation of the minigene construct; arrows indicate the primers for amplification of the insert. Middle: RT-PCR analysis of HEK293T cells transfected with minigene plasmids (WT and containing the variant c.999-556C>G) showing PE inclusion in the mutant (c.999-556C>G) construct but not in the wild-type (WT) construct. Numbers 1 and 2 denote technical replicates of RT-PCR. Bottom: fragment analysis demonstrating predominant PE-containing transcripts in the MUT construct and only the canonical transcript (Emp – empty) in the WT construct.

Treatment of fibroblasts with an NMD inhibitor increased the relative abundance of the pseudoexon-containing transcript in the heterozygous father’s cells by approximately 1,7-fold, although the increase did not restore the transcript to the ∼50% expected from a heterozygous genotype (Supplementary Figure S1).

A minigene assay independently confirmed the RNA findings (Figure 2B). The MUT construct generated predominantly the aberrant transcript containing the 98-nt PE (84.3%), together with a minor canonical transcript (15.5%), whereas the WT construct produced only the canonical transcript.

Collectively, these findings confirm that the c.999-556C>G variant induces aberrant PE inclusion and impairs *MFSD8* splicing, thereby disrupting the translation of a functional protein. We have therefore established the pathogenicity of this variant, and, given the concordant clinical phenotype, we consider it to be the causative mutation. Accordingly, all other variants identified by WGS were ruled out as unlikely to be disease-causing.

## Discussion

In this study, we established the molecular diagnosis of CLN7 disease in a patient with a severe progressive neurodegenerative phenotype by combining trio whole-genome sequencing with functional RNA analysis. WGS identified the previously unreported homozygous deep intronic *MFSD8* c.999-556C>G variant, which was subsequently shown to cause aberrant pseudoexon inclusion. This finding expands the mutational spectrum of *MFSD8* and highlights the diagnostic relevance of deep intronic variants, which may remain undetected by exon-focused sequencing approaches.

Importantly, analysis of patient-derived RNA provided direct evidence of aberrant splicing at the endogenous MFSD8 locus. Targeted RNA sequencing of fibroblasts demonstrated inclusion of the predicted 98-nt pseudoexon, resulting in a frameshift and premature termination codon, with the aberrant transcript accounting for 84% of the transcript molecules detected in the targeted amplicon in the proband. In the heterozygous father, the corresponding transcript represented 12% of the transcript molecules detected in the targeted amplicon. These values reflect relative isoform proportions within the analyzed RNA amplicons rather than absolute transcript abundance. This difference is compatible with partial degradation of the premature-termination-containing transcript by NMD, although the contribution of NMD could not be directly quantified because no informative heterozygous exonic variants were available for allele-specific analysis.

The minigene assay provided independent evidence that the observed splicing defect was directly attributable to the c.999-556C>G variant. The mutant construct reproduced the pseudoexon inclusion observed in patient-derived RNA, whereas the wild-type construct generated only the canonical transcript. The concordance between endogenous RNA analysis and the minigene assay strengthens the causal relationship between the deep intronic variant and the aberrant splicing event. The presence of a minor canonical transcript in the mutant construct indicates that the variant strongly promotes, but does not completely abolish, recognition of the canonical splice sites. However, the relative abundance of canonical transcripts in the minigene system cannot be directly extrapolated to the endogenous locus or used to infer residual MFSD8 protein function.

The molecular findings were consistent with the clinical presentation, which closely matched the characteristic phenotype of CLN7 disease, including childhood-onset epilepsy, progressive developmental regression, myoclonus, ataxia, dysarthria, and cerebral and cerebellar atrophy. Notably, despite severe neurological progression, no clinically significant retinal degeneration was observed at the time of evaluation. The absence of prominent retinal involvement in this patient further illustrates the clinical variability that may occur in CLN7 disease.

The identification of a defined pseudoexon-mediated splicing defect also has potential therapeutic implications. Personalized antisense oligonucleotide (ASO) therapy has previously been developed for an *MFSD8*-related CLN7 case, demonstrating the feasibility of correcting aberrant *MFSD8* splicing (Kim et al., 2019). The c.999-556C>G variant identified here produces a clearly defined and measurable splicing defect, providing a potential molecular endpoint for evaluating splice-correcting ASOs. Thus, functional characterization of deep intronic *MFSD8* variants may have implications beyond diagnostic classification and could provide a rationale for individualized therapeutic development.

## Conclusion

Our work expands the *MFSD8* mutational landscape and supplies functional data that confirm the pathogenicity of a novel splice-altering deep intronic variant. These findings also underscore the diagnostic power of pairing WGS with RNA analysis for solving genetically undiagnosed rare neurogenetic diseases.

## Data Availability

The datasets presented in this study can be found in online repositories. The names of the repository/repositories and accession number(s) can be found in the article/Supplementary Material.

## Supplementary material

SUPPLEMENTARY FIGURE S1 Sashimi plot generated in the IGV genome browser. The plot illustrates PE inclusion within intron 9 of *MFSD8* across cDNA samples obtained from fibroblast cultures of the father (treated with cycloheximide (CHX) or untreated (UT).) and a healthy donor.
