# Supplementary material for: Case Report: A novel deep intronic MFSD8 splice-altering variant causes CLN7 disease through pseudoexon inclusion
Source: Front Genet. 2026 Oct 20;17:1980180. doi: 10.3389/fgene.2026.1980180 (PMC13727524; doi:10.3389/fgene.2026.1980180)
Supplement: Supplementary file 1 [file Supplementaryfile1.docx]

**Supplementary materials**

**Materials and Methods**

**Subjects**

The proband and her parents underwent clinical evaluation, genetic testing, and genetic counseling at the Research Centre for Medical Genetics (RCMG), Moscow, Russia. Skin fibroblast cultures were established from the proband and her heterozygous father for RNA analysis. A fibroblast culture from an unrelated healthy individual served as a control. All cell lines, including a healthy control, were sourced from and are stored at the Moscow branch of the biobank “All-Russian Collection of Biological Samples of Hereditary Diseases. Written informed consent was obtained from the patient's legal representatives. The study was approved by the Institutional Review Board of the Research Centre for Medical Genetics.

**Whole genome sequencing**

Trio whole-genome sequencing (WGS) was performed for the proband and both parents. PCR-free libraries were prepared according to the manufacturer's protocol and sequenced as 2 × 150 bp paired-end reads on a DNBSEQ-T7 platform (MGI) to a mean genome coverage of approximately 30×. Reads were aligned to the hg38 reference genome using BWA (v0.7.17). Single-nucleotide variants and small insertions/deletions were identified using DeepVariant, while structural variants were analyzed with Manta (v1.6.0). Variants were annotated using the certified NGS-DATA-OMICS software (certificate No. RU 2025685783). Population allele frequencies were obtained from the 1000 Genomes Project and gnomAD (v3.1.2 and v4.0.0). Variant interpretation was performed according to the ACMG/AMP guidelines.

**RNA analysis**

Fibroblasts from the proband’s father and healthy donors (controls) were cultured in high-glucose DMEM supplemented with 10% fetal bovine serum at 37°C in a 5% CO₂ atmosphere.

Total RNA was isolated from cultured skin fibroblasts using the MagPure Universal RNA Kit (Magen) on an AutoPure S32 automated workstation. First-strand cDNA synthesis was performed using oligo(dT) primers and the Reverse Transcription System (Dialat, Russia). cDNA quality was verified by quantitative PCR of the B2M housekeeping gene.

To evaluate the effect of the *MFSD8* variant NM_001371596.2:c.999-556C>G on pre-mRNA splicing, a fragment spanning exons 6–12 was amplified by RT-PCR using the following primers: 5′-AGGTGTGACATGGGATGTGAT-3′, 5′-GGTCCCCAGTGAGCATACAC-3′. The RT-PCR amplicons obtained were subsequently analyzed by targeted NGS.

**NGS of targeted RT-PCR products**

Sequencing libraries were prepared using the SG GM Kit (Raissol) and sequenced on the FASTASeq platform as 2 × 150 bp paired-end reads, achieving >129,000× mean coverage across the target region. Sequencing reads were quality-controlled using FastQC (v0.12.1), aligned to the hg38 reference genome with STAR (v2.7.11b), and splice junctions were visualized using Sashimi plots in IGV.

**Nonsense-mediated decay inhibition in fibroblast cultures**

Fibroblasts from the proband’s father and healthy donor (control) were seeded at a density of 3 × 10⁵ cells per well in 6-well plates 24 h before treatment. Cells were cultured in high-glucose DMEM supplemented with 10% fetal bovine serum at 37°C in a 5% CO₂ atmosphere. At the time of treatment, cell confluence was approximately 80%.

Two culture conditions were used: control medium to assess the potential effect of the solvent untreated - UT) and medium supplemented with cycloheximide (+CHX). CHX was added at a final concentration of 50 μg/mL, corresponding to a 1:1000 dilution of the stock solution. Fibroblasts from the proband’s father and control fibroblasts were incubated in parallel either in the presence of CHX or in control medium without CHX, containing DMSO at a 1:1000 dilution, for 24 h at 37°C in a 5% CO₂ atmosphere.

After incubation, the cells were visually examined under a microscope to confirm the absence of apparent toxic effects. The culture medium was then removed, and the cells were lysed directly in the wells of the 6-well plate using RLT buffer (Magen) supplemented with β-mercaptoethanol. Samples were immediately frozen and stored at -80°C until subsequent total RNA extraction.

**Minigene analysis**

Genomic DNA was extracted from peripheral blood samples obtained from the proband and a healthy donor. A genomic fragment encompassing *MFSD8* intron 8 and the candidate PE was amplified from the proband (mutant, MUT) and control (wild-type, WT) genomic DNA using the following primers: forward, 5′-cagaattctggagctcgagGCAGATGACATGATTGCTGTATTTCT-3′; reverse, 5′-attaaggagtgtattaagcttTCCACTAAAAATAACCAGGACTCCT-3′. PCR products were cloned into the pSpl3-Flu splicing reporter vector using Gibson assembly.

WT and MUT minigene constructs were transfected separately into HEK293T cells using Genject-39 reagent (Molekta, Russia) according to the manufacturer's instructions. Forty-eight hours after transfection, total RNA was isolated and reverse transcribed as described above. RT-PCR was performed using plasmid-specific primers flanking the vector exons (forward, 5′-ACAAAGAGACCTACGTCGAGCA-3′; reverse, 5′-/6-FAM/-AGCTCGATCAGCACGGGCACGAT-3′). Amplification products were analyzed by fluorescent fragment analysis to quantify the relative abundance of canonical and aberrant splice isoforms, with agarose gel electrophoresis used for qualitative visualization.
